# Comparisons of three novel markers for insulin resistance to predict incident cardiovascular disease: a Korean cohort study from three different regions

**DOI:** 10.1186/s40001-025-02374-0

**Published:** 2025-03-20

**Authors:** Ha Eun Ryu, Yong Jae Lee, Byoungjin Park, Dong Hyuk Jung

**Affiliations:** 1 Department of Family Medicine, Yongin Severance Hospital, 363 Dongbaekjukjeondae-Ro, Giheung-Gu, Yongin-Si, Gyeonggi-Do 16995 Republic of Korea; 2 Department of Family Medicine, Yonsei University College of Medicine, Seoul, 03722 Republic of Korea; 3 3Department of Family Medicine, Gangnam Severance Hospital, Seoul, 06273 Republic of Korea

**Keywords:** Cardiovascular disease, Triglyceride–glucose index, Atherogenic index of plasma, Metabolic score for insulin resistance, Cohort study, Koreans

## Abstract

**Background:**

Cardiovascular disease (CVD) is a crucial human health challenge. Previous studies have shown an association between CVD and the triglyceride–glucose (TyG) index, atherogenic index of plasma (AIP), and metabolic score for insulin resistance (METS–IR). However, a comparison of these novel markers for predicting CVD is not well known. Therefore, we aimed to assess the value of TyG, AIP, and METS–IR in predicting the incidence of CVD in three large cohorts of Korean adults.

**Methods:**

Data from 28 437 participants in the Korean Genome and Epidemiology Study (KoGES) and Korea Health Insurance Review and Assessment (HERAS–HIRA) were assessed. The participants were divided into four groups according to the quartiles of TyG index: ln ([triglyceride × fasting plasma glucose]/2), AIP calculated as log (triglyceride/high-density lipoprotein cholesterol), and METS–IR index: (ln ([2 × fasting plasma glucose] + triglyceride) × body mass index)/(ln [high-density lipoprotein cholesterol–cholesterol]). We prospectively assessed the hazard ratios (HRs) with 95% confidence intervals (CIs) for CVD using multivariate Cox proportional hazard regression models after adjusting for potential confounding variables.

**Results:**

During the follow-up period, 987 participants (3.5%) developed CVD. Compared with the referent first quartiles, the highest TyG index, AIP, and METS–IR quartiles, with HRs of 1.73 (95% CI 1.41–2.12), 1.47 (95% CI 1.19–1.80), and 2.61 (95% CI 1.83–3.72), respectively, significantly predicted future CVD, after adjusting for age, sex, and body mass index. When comparing the three biomarkers for insulin resistance, the TyG index and METS–IR showed similar predictive values, whereas AIP had a lower significance in predicting CVD.

**Conclusions:**

Based on the current findings, novel surrogate markers of insulin resistance, particularly METS–IR and TyG index, may help predict the risk of CVD in Koreans.

## Background

Cardiovascular disease (CVD) poses considerable challenges to human health. Based on findings from the Global Burden of Disease (GBD) 2016 study, CVDs have a remarkable impact on the health burden experienced by both men and women. CVDs constituted 20% of the total burden in women and 24% in men. In addition, the ranking of CVDs in terms of the total burden remained unchanged between 2000 and 2016, with most CVDs experiencing an increase in total burden over time [1, 2].

The occurrence of CVD is closely linked to an unhealthy lifestyle and the presence of multiple coexisting conditions, such as metabolic syndrome, high blood pressure, dyslipidemia, and diabetes [3]. However, these risk factors for CVD are preventable [4]. Consequently, there has been a demand for early detection and intervention among high-risk groups, leading to several studies on predictive indicators [5].

The role of atherosclerosis has been emphasized in the traditional understanding of CVD pathophysiology. Atherosclerosis is a chronic, immune-inflammatory disease of medium-sized and large arteries characterized by the accumulation of lipids and the involvement of endothelial cells, leukocytes, and intimal smooth muscle cells in its pathogenesis [6]. Therefore, the prediction of CVD has traditionally relied on lipid parameters, such as low-density lipoprotein cholesterol (LDL-C), high-density lipoprotein cholesterol (HDL-C), and triglyceride (TG) levels. However, these markers offer limited information and fail to accurately reflect the progression of atherosclerotic lesions or the prognosis of patients with CVD [7–11]. Thus, it has become apparent that relying solely on traditional markers has limitations in predicting CVD. Consequently, there is an ongoing demand for new markers to address these limitations.

Insulin resistance (IR) has been consistently associated with an increased risk of coronary artery disease (CAD) in previous studies [12–15]. Novel IR markers have been proposed for this purpose. Examples of such novel markers include the triglyceride–glucose (TyG) index, atherogenic index of plasma (AIP), and metabolic score for insulin resistance (METS–IR). In previous studies, each novel marker has demonstrated meaningful results in predicting CVD [16–21]. However, there is a lack of knowledge regarding comparing these novel markers in predicting CVD.

Therefore, this study aimed to evaluate the predictive value of the TyG index, AIP, and METS–IR for incident CVD in a large cohort of Korean adults. By investigating the performance of these markers, we hope to enhance our understanding of their utility in predicting CVD and to identify superior markers for clinical risk assessment.

## Materials and methods

### Study design and participants

**Fig. 1:**
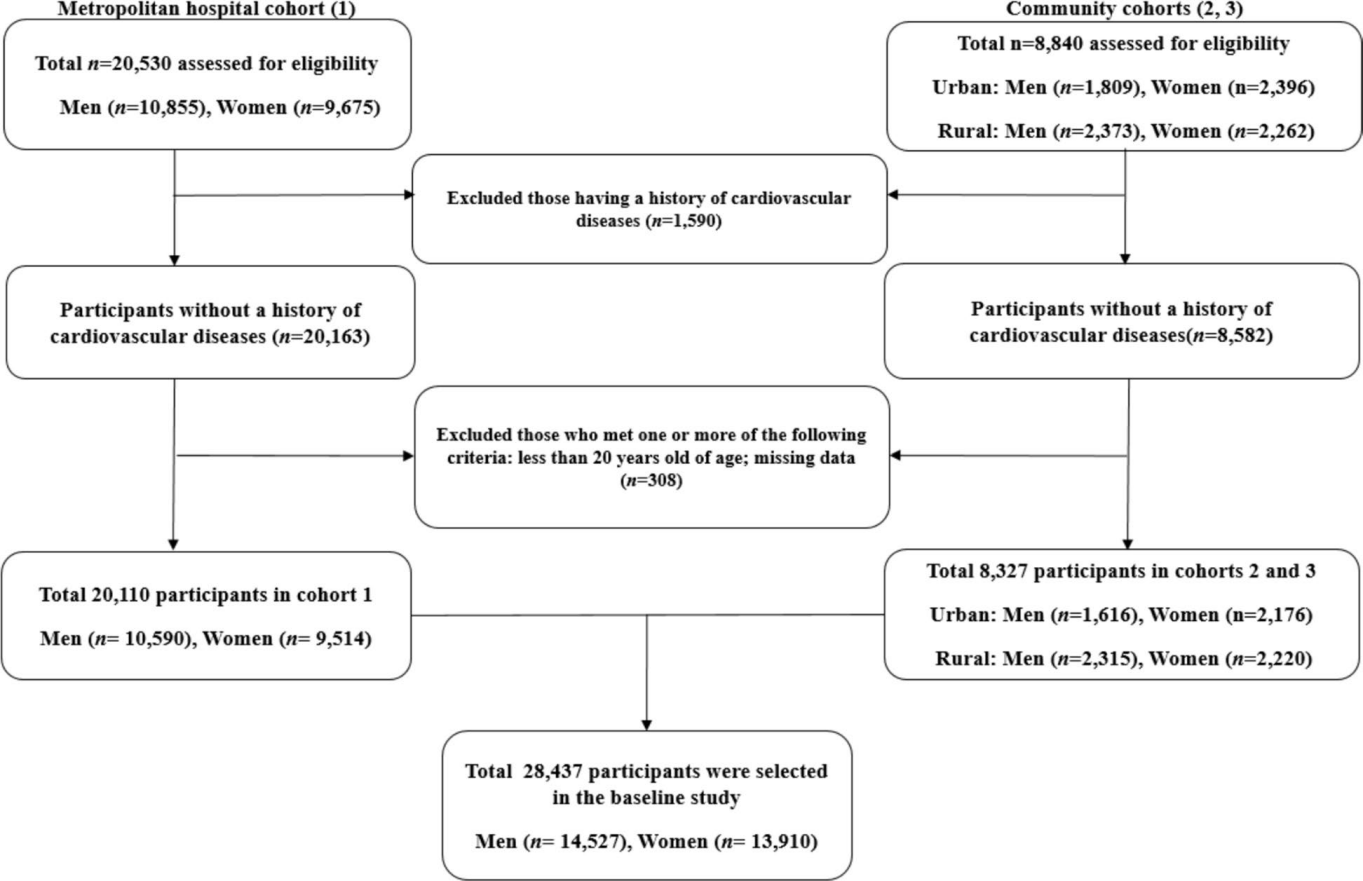
Flowchart for the selection of study participants

Two types of data were used in this study. The first data set was obtained from the Korean Genome and Epidemiology Study (KoGES), which has been extensively described in its design and methodology. This study aimed to evaluate the prevalence, incidence, and risk factors of chronic degenerative disorders, including diabetes, hypertension, osteoporosis, and cardiovascular disease. The KoGES cohort comprised 10,030 participants residing in both urban (Ansan) and rural (Ansung) areas. The participants were recruited during a baseline survey conducted between 2001 and 2002. Subsequently, biennial surveys were conducted until the sixth follow-up visit (2013–2014) over 12 years. Second, we used data from the Health Risk Assessment Study and Korea Health Insurance Review and Assessment (HERAS–HIRA) to explore surrogate markers for CVD among Koreans. Briefly, the cohort consisted of 20,530 participants who visited the Health Promotion Center at Yonsei University Gangnam Severance Hospital for health examinations. Most participants resided in the metropolitan Gangnam area of Seoul. The baseline survey for this cohort was conducted between November 2006 and June 2010, and participants were assessed over 50 months from enrollment. Participants meeting any of the following criteria were excluded: previous diagnosis of CVD, age < 20 years, missing data. Finally, 28,437 participants without CVD were included in this study (Fig. 1). Informed consent was obtained from all the eligible participants. This study was approved by the Institutional Review Board (IRB) of the Yongin Severance Hospital (IRB number 9–2020-0018).

### Data collection

Each participant completed a comprehensive questionnaire that captured information about their lifestyle and medical history. Smoking status was categorized as never smoked, ex-smoker, or current smoker. Moderate alcohol consumption was defined as the consumption of > 140 g of alcohol per week based on the frequency of alcohol consumption reported by the participants [22]. Bodyweight and height measurements were taken, while participants wore light indoor clothing and no shoes, with a precision of 0.1 kg and 0.1 cm, respectively. Body mass index (BMI) was calculated as weight divided by height in meters squared (kg/m2). Systolic blood pressure (SBP) and diastolic blood pressure (DBP) were measured in the sitting position after 10 min of rest using a standard mercury sphygmomanometer (Baumanometer; W.A. Baum Co Inc., Copiague, NY, USA). Mean arterial pressure was derived from the SBP and DBP values and weighted as 1/3 SBP and 2/3 DBP [23]. After a 12-h overnight fast, blood samples were collected from the participants through the antecubital vein. Concentrations of total cholesterol, triglycerides, HDL cholesterol, and plasma glucose were measured enzymatically using a Chemistry Analyzer (Hitachi 7600, Tokyo, Japan by August 2002 and ADVIA 1650, Siemens, Tarrytown, NY, September 2002). C-reactive protein (CRP) concentration was measured using an immunoradiometric assay (ADVIA 1650, Siemens, Tarrytown, NY, USA). Type 2 diabetes was defined by a fasting plasma glucose level of at least 126 mg/dl or the current use of diabetes medication. The TyG index was determined using the following formula: ln ([triglyceride × fasting plasma glucose]/2). The formula for calculating AIP was log (triglyceride/high-density lipoprotein cholesterol). METS–IR was calculated as ln ([2 × fasting plasma glucose] + triglyceride) × BMI)/(ln [HDL-cholesterol].

### Study outcomes

The primary outcome of this study, as previously described, was CVD, which mainly encompasses angina pectoris (ICD-10 code I20) or acute myocardial infarction (ICD-10 code I21) that occurred after participant enrollment in the study. To establish baseline and post-survey outcomes, we utilized a personal 13-digit identification number assigned to each participant by the Korean Health Insurance Review and Assessment Service (HIRA) between November 1, 2006, and December 31, 2010. For the KoGES cohort, the self-reported CVD status of the participants was obtained through a questionnaire.

### Statistical analysis

We divided the TyG index, AIP, and METS–IR values into quartiles and compared the clinical characteristics at the baseline, respectively. All data are presented as means with standard deviation or percentage. According to the markers of each IR quartile, the baseline characteristics of the study population were compared using a chi-squared test for categorical variables (male sex, current smoker, alcohol drinking, hypertension medication, diabetes medication, and dyslipidemia medication) and analysis of variance (ANOVA) model for continuous variables (age, BMI, SBP, DBP, mean arterial pressure, fasting plasma glucose, total cholesterol, triglyceride, HDL-cholesterol, C-reactive protein, and estimated glomerular filtration rate). In multivariate analysis, after setting the lowest group of each IR value quartile as the reference group, hazard ratios (HRs) and 95% confidence intervals (CIs) for new-onset CVD were calculated using the Cox proportional hazards regression model after adjusting for potential confounding variables. We have also analyzed the same way, using the TyG index, AIP, and METS-IR as continuous variables in their association with CVD. We used pairwise comparisons of receiver-operating characteristic (ROC) curves and concordance (C) statistics to assess the ability of a risk factor to predict CVD with the identification of the cutoff value for each IR marker. All analyses were performed using the SAS version 9.4 software (SAS Institute Inc., Cary, NC, USA). All statistical tests were two-sided, and *P* values < 0.05 were considered statistically significant.

## Results

### Baseline characteristics of study populations

Overall, 28,437 participants (51.1% males) were enrolled in the current study. Tables 1, 2 and 3 demonstrate the participants’ baseline clinical, demographic, and anthropometric features according to surrogate markers related to IR, such as the TyG index, AIP, and METS–IR, respectively. The mean age, TyG index, AIP, and METS–IR were 47.6 ± 10.7 years, 8.57 ± 0.61,—0.01 ± 0.30, and 33.22 ± 7.10, respectively.

**Table 1:** Baseline characteristics of the study population according to the TyG index quartiles

Characteristics	Overall (*n* = 28,437)	Q1 (*n* = 7114)	Q2 (*n* = 7177)	Q3 (*n* = 7000)	Q4 (*n* = 7146)	*P-value*^a^	*Post hoc*^b^
TyG index	8.57 ± 0.61	≤ 8.13	8.14–8.52	8.53–8.94	≥ 8.95		
Age (years)	47.6 ± 10.7	44.2 ± 10.6	47.4 ± 10.8	49.2 ± 10.5	49.6 ± 10.0	< 0.001	a,b,c,d,e
Body mass index (kg/m^2^)	22.6 ± 3.9	21.3 ± 3.1	22.3 ± 3.5	23.1 ± 4.0	23.6 ± 4.4	< 0.001	a,b,c,d,e,f
Male sex (%)	51.1	30.3	45.8	57.8	70.6	< 0.001	–
Current smoker (%)	22.8	14.7	21.1	25.3	29.9	< 0.001	–
Alcohol drinking (%)	34.5	27.8	32.1	35.2	42.9	< 0.001	–
Systolic blood pressure (mmHg)	121.9 ± 16.8	115.0 ± 14.9	120.2 ± 16.3	124.4 ± 16.5	128.3 ± 16.6	< 0.001	a,b,c,d,e,f
Diastolic blood pressure (mmHg)	77.4 ± 10.9	72.3 ± 9.7	76.1 ± 10.4	79.2 ± 10.5	81.9 ± 10.5	< 0.001	a,b,c,d,e,f
Mean arterial pressure (mmHg)	92.2 ± 12.3	86.6 ± 10.9	90.8 ± 11.8	94.2 ± 11.8	97.4 ± 11.8	< 0.001	a,b,c,d,e,f
Fasting plasma glucose (mg/dl)	94.0 ± 20.5	85.6 ± 8.2	90.1 ± 9.9	93.5 ± 13.0	106.6 ± 33.2	< 0.001	a,b,c,d,e,f
Total cholesterol (mg/dl)	192.1 ± 35.2	176.5 ± 30.4	187.6 ± 31.5	197.4 ± 33.7	206.8 ± 37.6	< 0.001	a,b,c,d,e,f
Triglyceride (mg/dl)	133.8 ± 97.0	61.5 ± 11.8	93.1 ± 13.4	133.3 ± 21.7	247.1 ± 130.2	< 0.001	a,b,c,d,e,f
HDL-cholesterol (mg/dl)	52.1 ± 12.6	60.0 ± 12.5	54.4 ± 11.9	49.4 ± 10.7	44.4 ± 9.3	< 0.001	a,b,c,d,e,f
C-reactive protein (mg/L)	1.1 ± 3.4	0.9 ± 3.0	1.0 ± 3.5	1.2 ± 3.8	1.2 ± 3.2	< 0.001	b,c,e
eGFR (mL/min/1.73 m^2^)	86.8 ± 17.4	88.5 ± 17.2	87.3 ± 17.4	86.0 ± 17.1	85.5 ± 17.5	< 0.001	a,b,c,d,e
Hypertension medication (%)	11.0	4.3	7.4	11.8	20.6	< 0.001	–
Diabetes medication (%)	11.4	3.9	8.2	12.8	20.7	< 0.001	–
Dyslipidemia medication (%)	2.3	0.9	1.6	2.6	4.1	< 0.001	–

^a^P values were calculated using 1-way ANOVA or chi-squared test

^b^Post hoc analysis with the Bonferroni method: a, Q1 vs. Q2; b, Q1 vs. Q3; c, Q1 vs. Q4; d, Q2 vs. Q3; e, Q2 vs. Q4; and f, Q3 vs. Q4

TyG index: triglyceride-glucose index; HDL-C: High-density lipoprotein cholesterol; eGFR: estimated glomerular filtration rate

**Table 2:** Baseline characteristics of the study population according to the AIP quartiles

Characteristics	Overall (*n* = 28,437)	Q1 (*n* = 7100)	Q2 (*n* = 7069)	Q3 (*n* = 7167)	Q4 (*n* = 7101)	*P value*^a^	*Post hoc*^b^
AIP	− 0.01 ± 0.30	≤ − 0.23	− 0.22–− 0.03	− 0.02–0.19	≥ 0.20		
Age (years)	47.6 ± 10.7	44.6 ± 10.8	47.7 ± 10.8	49.0 ± 10.5	49.1 ± 10.1	< 0.001	a,b,c,d,e
Body mass index (kg/m^2^)	22.6 ± 3.9	21.3 ± 3.0	22.4 ± 3.5	23.2 ± 4.0	23.4 ± 4.4	< 0.001	a,b,c,d,e,f
Male sex (%)	51.1	28.6	45.9	59.3	70.4	< 0.001	–
Current smoker (%)	22.8	15.1	20.6	26.4	28.8	< 0.001	–
Alcohol drinking (%)	34.5	29.2	32.0	35.7	41.0	< 0.001	–
Systolic blood pressure (mmHg)	121.9 ± 16.8	116.1 ± 15.5	120.7 ± 16.4	124.1 ± 16.6	126.9 ± 16.7	< 0.001	a,b,c,d,e,f
Diastolic blood pressure (mmHg)	77.4 ± 10.9	72.9 ± 10.1	76.4 ± 10.5	78.9 ± 10.7	81.3 ± 10.6	< 0.001	a,b,c,d,e,f
Mean arterial pressure (mmHg)	92.2 ± 12.3	87.3 ± 11.4	91.2 ± 11.9	94.0 ± 12.0	96.5 ± 11.9	< 0.001	a,b,c,d,e,f
Fasting plasma glucose (mg/dl)	94.0 ± 20.5	88.7 ± 13.8	92.2 ± 17.3	95.6 ± 21.4	99.4 ± 25.9	< 0.001	a,b,c,d,e,f
Total cholesterol (mg/dl)	192.1 ± 35.2	182.2 ± 31.5	187.8 ± 33.2	196.0 ± 35.2	202.2 ± 37.4	< 0.001	a,b,c,d,e,f
Triglyceride (mg/dl)	133.8 ± 97.0	63.2 ± 14.3	93.1 ± 17.9	131.7 ± 26.2	247.0 ± 130.4	< 0.001	a,b,c,d,e,f
HDL-cholesterol (mg/dl)	52.1 ± 12.6	64.7 ± 11.6	54.3 ± 9.3	48.0 ± 8.0	41.3 ± 7.1	< 0.001	a,b,c,d,e,f
C-reactive protein (mg/L)	1.1 ± 3.4	0.8 ± 2.6	1.0 ± 3.2	1.2 ± 4.5	1.2 ± 2.9	< 0.001	a,b,c,d,e
eGFR (mL/min/1.73 m^2^)	86.8 ± 17.4	88.0 ± 16.9	87.2 ± 17.5	86.3 ± 17.2	85.8 ± 17.7	< 0.001	a,b,c,d,e
Hypertension medication (%)	11.0	5.4	9.1	12.8	16.8	< 0.001	
Diabetes medication (%)	11.4	4.6	9.3	13.5	18.2	< 0.001	–
Dyslipidemia medication (%)	2.3	1.2	2.1	2.5	3.3	< 0.001	–

^a^P values were calculated using 1-way ANOVA or chi-squared test

^b^Post hoc analysis with the Bonferroni method: a, Q1 vs. Q2; b, Q1 vs. Q3; c, Q1 vs. Q4; d, Q2 vs. Q3; e, Q2 vs. Q4; and f, Q3 vs. Q4

AIP: Atherogenic index of plasma; HDL-C: High-density lipoprotein cholesterol; eGFR: estimated glomerular filtration rate

**Table 3:** Baseline characteristics of the study population according to the METS-IR quartiles

Characteristics	Overall (*n* = 28,437)	Q1 (*n* = 7111)	Q2 (*n* = 7105)	Q3 (*n* = 7117)	Q4 (*n = *7104)	*P value*^a^	*Post hoc*^b^
METS-IR	33.22 ± 7.10	≤ 27.84	27.85–32.67	32.68–37.92	≥ 37.93		
Age (years)	47.6 ± 10.7	42.4 ± 8.2	47.1 ± 10.0	49.9 ± 10.6	50.9 ± 11.6	< 0.001	a,b,c,d,e,f
Body mass index (kg/m^2^)	22.6 ± 3.9	18.0 ± 2.2	21.5 ± 1.7	23.8 ± 1.7	27.0 ± 2.5	< 0.001	a,b,c,d,e,f
Male sex (%)	51.1	38.5	41.8	59.9	64.2	< 0.001	–
Current smoker (%)	22.8	21.0	19.2	25.9	25.0	< 0.001	–
Alcohol drinking (%)	34.5	42.4	32.2	31.5	31.7	< 0.001	–
Systolic blood pressure (mmHg)	121.9 ± 16.8	113.6 ± 15.0	119.0 ± 15.9	124.7 ± 15.4	130.5 ± 15.9	< 0.001	a,b,c,d,e,f
Diastolic blood pressure (mmHg)	77.4 ± 10.9	73.8 ± 11.2	75.3 ± 10.6	78.6 ± 10.1	81.9 ± 9.9	< 0.001	a,b,c,d,e,f
Mean arterial pressure (mmHg)	92.2 ± 12.3	87.0 ± 11.7	89.8 ± 11.8	94.0 ± 11.3	98.1 ± 11.3	< 0.001	a,b,c,d,e,f
Fasting plasma glucose (mg/dl)	94.0 ± 20.5	88.3 ± 14.1	91.4 ± 17.7	95.0 ± 19.2	101.2 ± 26.6	< 0.001	a,b,c,d,e,f
Total cholesterol (mg/dl)	192.1 ± 35.2	188.5 ± 34.0	188.3 ± 34.1	193.0 ± 35.0	198.5 ± 36.9	< 0.001	b,c,d,e,f
Triglyceride (mg/dl)	133.8 ± 97.0	99.5 ± 63.9	107.7 ± 73.7	132.0 ± 75.5	196.1 ± 129.2	< 0.001	a,b,c,d,e,f
HDL-cholesterol (mg/dl)	52.1 ± 12.6	59.4 ± 14.0	55.6 ± 11.5	49.7 ± 9.5	43.5 ± 8.5	< 0.001	a,b,c,d,e,f
C-reactive protein (mg/L)	1.1 ± 3.4	0.5 ± 1.9	0.9 ± 3.0	1.3 ± 4.1	1.6 ± 4.1	< 0.001	a,b,c,d,e,f
eGFR (mL/min/1.73 m^2^)	86.8 ± 17.4	91.7 ± 19.0	87.4 ± 17.1	84.8 ± 16.1	83.4 ± 15.9	< 0.001	a,b,c,d,e,f
Hypertension medication (%)	11.0	4.0	7.8	13.2	19.1	< 0.001	–
Diabetes medication (%)	11.4	9.5	8.7	11.7	15.8	< 0.001	–
Dyslipidemia medication (%)	2.3	0.7	1.5	2.8	4.2	< 0.001	–

^a^P values were calculated using 1-way ANOVA or chi-squared test

^b^Post hoc analysis with the Bonferroni method: a, Q1 vs. Q2; b, Q1 vs. Q3; c, Q1 vs. Q4; d, Q2 vs. Q3; e, Q2 vs. Q4; and f, Q3 vs. Q4

METS-IR: metabolic score for insulin resistance; HDL-C: High-density lipoprotein cholesterol; eGFR: estimated glomerular filtration rate

The group with the fourth TyG index, AIP, and METS–IR quartiles showed the highest mean values of BMI, mean blood pressure, fasting plasma glucose, total cholesterol, and triglycerides; however, the mean HDL-cholesterol levels and estimated glomerular filtration rate (eGFR) were highest in the lowest TyG index, AIP, and METS–IR quartiles. Those in the highest quartiles for all IR markers were likely to have a history of smoking and medications for hypertension, diabetes, and dyslipidemia. The proportion of alcohol consumption increased according to the TyG index and AIP quartiles; however, the METS–IR quartiles did not exhibit significant trends.

### HRs for CVD and comparison among three IR markers

Eventually, 987 individuals (3.5%, 987/28,437) developed CVD during the follow-up period. The CVD incidence rate (per 1000 person-years) was positively correlated with the TyG index, AIP, and METS–IR quartiles. Table 4 shows the multivariate Cox proportional hazards regression analysis results for predicting CVD according to the TyG index, AIP, and METS–IR quartiles. Compared with the first reference TyG index, AIP, and METS–IR, the HRs of incident CVD for the second, third, and fourth quartiles increased gradually after adjusting for age, sex, and BMI. Similarly, positive associations were observed in the TyG index and METS–IR quartiles after adjusting for smoking status, alcohol intake, mean arterial blood pressure, total cholesterol, eGFR, C-reactive protein, hypertension medication, diabetes medication, dyslipidemia medication, and diabetes mellitus. AIP quartiles did not exhibit any significant trends, but AIP levels as a continuous variable showed a positive association with CVD.

**Table 4:** HRs and 95% CIs for cardiovascular disease using the TyG index, AIP, and METS-IR as quartiles and continuous variables

TyG index quartile	Q1 (≤ 8.13, *n* = 7114)	Q2 (8.14–8.52, *n* = 7177)	Q3 (8.53–8.94, *n* = 7000)	Q4 (≥ 8.95, *n* = 7146)	*P* for trend
New cases of CVD, *n*	126	215	271	375	
Mean follow-up, years	3.5 ± 2.9	3.8 ± 3.2	3.9 ± 3.3	4.0 ± 3.3	
Person-years of follow-up	24,741	27,178	27,432	28,830	
Incidence rate/1000 person-years	5.1	7.9	9.9	13.0	
Model 1 HR (95% CI)	1.00 (reference)	1.21 (0.97–1.51)	1.35 (1.09–1.67)	1.73 (1.41–2.12)	< 0.001
*P* value	–	0.089	0.005	0.021	
Model 2 HR	1.00 (reference)	1.22 (0.97–1.54)	1.24 (0.99–1.56)	1.38 (1.10–1.74)	0.054
*P* value	–	0.090	0.061	0.006	
AIP quartile	Q1 (≤ − 0.23, *n* = 7100)	Q2 (− 0.22–− 0.03, *n* = 7069)	Q3 (− 0.02–0.19, *n* = 7167)	Q4 (≥ 0.20, *n* = 7101)	
New cases of CVD, *n*	129	219	290	349	
Mean follow-up, years	3.3 ± 2.8	3.8 ± 3.1	4.0 ± 3.3	4.2 ± 3.4	
Person-years of follow-up	23,430	26,537	28,409	29,804	
Incidence rate/1000 person-years	5.5	8.3	10.2	11.7	
Model 1 HR (95% CI)	1.00 (reference)	1.17 (0.94–1.46)	1.30 (1.05–1.60)	1.47 (1.19–1.80)	0.001
*P* value	–	0.150	0.015	< 0.001	
Model 2 HR	1.00 (reference)	1.10 (0.88–1.38)	1.14 (0.92–1.42)	1.20 (0.97–1.49)	0.412
*P* value	–	0.403	0.239	0.100	
METS-IR quartile	Q1 (≤ 27.84, *n* = 7111)	Q2 (27.85–32.67, *n* = 7105)	Q3 (32.68–37.92, n = 7117)	Q4 (≥ 37.93, *n* = 7104)	
New cases of CVD, *n*	121	208	288	370	
Mean follow-up, years	1.7 ± 0.8	2.3 ± 0.9	2.8 ± 1.0	3.2 ± 1.0	
Person-years of follow-up	35,031	25,886	23,903	23,360	
Incidence rate/1000 person-years	3.5	8.0	12.0	15.8	
Model 1 HR (95% CI)	1.00 (reference)	1.78 (1.38–2.29)	2.12 (1.59–2.83)	2.61 (1.83–3.72)	< 0.001
*P* value	–	< 0.001	< 0.001	< 0.001	
Model 2 HR	1.00 (reference)	1.72 (1.33–2.23)	1.85 (1.37–2.50)	1.97 (1.36–2.85)	< 0.001
*P* value	–	< 0.001	< 0.001	< 0.001	

Continuous variables	TyG index	AIP	METS-IR
Model 1 HR (95% CI)	1.43 (1.29–1.58)	1.73 (1.40–2.15)	1.06 (1.04–1.08)
*P value*	< 0.001	< 0.001	< 0.001
Model 2 HR (95% CI)	1.20 (1.07–1.35)	1.34 (1.07–1.69)	1.03 (1.01–1.05)
*P value*	0.002	0.012	0.001

TyG index: triglyceride-glucose index; AIP: Atherogenic index of plasma; METS-IR: metabolic score for insulin resistance; HR: hazard ratio; CI: confidence interval

Model 1: adjusted for age, sex, and body mass index

Model 2: adjusted for age, sex, body mass index, smoking status, alcohol intake, mean arterial blood pressure, total cholesterol, eGFR, C-reactive protein, hypertension medication, diabetes medication, dyslipidemia medication, and diabetes mellitus

Next, we compared the receiver operating characteristic (ROC) curve to compare the power of the predictive ability of CVD among the three markers for IR. A comparison of the TyG index with AIP showed that TyG was superior to AIP in predicting new-onset CVD (P = 0.017)**.** However, comparisons between the other variables did not show superiority in predicting CVD. The C-index, sensitivity, and specificity were 0.617 (95% CI 0.611–0.623), 80.7%, and 36.5% for the TyG index; 0.608 (95% CI 0.602–0.614), 68.6%, and 47.3% for AIP; and 0.618 (95% CI 0.612–0.623), 77.8%, and 40.8% for METS–IR, respectively (Table 5).

**Table 5:** TyG index vs. AIP vs. METS-IR for predicting cardiovascular disease

	Pairwise comparison of C-index			Ability to classify cardiovascular disease				
	Difference	95% CI	*P* value	Sensitivity (%)	Specificity (%)	Cutoff value	C-index	95% CI
TyG index vs. AIP	0.009	0.002 to 0.017	0.017					
TyG index vs. METS-IR	0.007	− 0.018 to 0.019	0.940					
AIP vs. METS-IR	0.010	− 0.007 to 0.027	0.259					
TyG index				80.7	36.5	> 8.30	0.617	0.611–0.623
AIP				68.6	47.3	> − 0.05	0.608	0.602–0.614
METS-IR				77.8	40.8	> 30.79	0.618	0.612–0.623

TyG index: triglyceride-glucose index; AIP: atherogenic index of plasma; METS-IR: metabolic score for insulin resistance

## Discussion

In this study, we examined the individual predictive associations of three surrogate markers of IR –the TyG index, AIP, and METS–IR–with the occurrence of CVD. In addition, we compared these markers as predictors of CVD. The results revealed that all the markers were positively associated with CVD incidence. When comparing the markers, the TyG index and METS–IR showed similar predictive values, whereas AIP was less significant in predicting CVD than the TyG index.

Atherosclerosis, the primary driver of CVD pathogenesis, is characterized by an imbalance in lipid removal and deposition, leading to a gradual buildup of lipids within arterial vessels [24]. In addition, the accumulation and oxidation of lipids in arteries, along with the formation and progression of fatty streaks and atherosclerotic lesions, accelerate the development and complications of atherosclerosis [25–28]. Previous studies have highlighted the significance of triglycerides and cholesterol esters as crucial circulating lipids implicated in atherosclerosis [29, 30]. Besides, IR is known to advance the progression of atherosclerosis by interfering with lipid metabolism and eliciting endothelial dysfunction [27, 28, 31].

IR, characterized by the diminished sensitivity of insulin-targeting tissues to normal insulin levels, hampers the disposal of insulin-mediated glucose and consequently triggers compensatory hyperinsulinemia [32–35]. Insulin plays a vital role in glucose and lipid metabolism by exerting its effects on multiple target tissues, including the liver, skeletal muscle, adipose tissue, endothelium, and vasculature [35]. Numerous previous studies have provided evidence for a relationship between IR and both the incidence of CVD [12–15, 32, 34] and its risk factors, such as hypertension [36], type 2 diabetes mellitus (T2DM) [37], nonalcoholic fatty liver disease [38], and obesity [39].

Considering this association, the evaluation of IR has emerged as a crucial aspect of predicting and managing CVD. Various surrogate markers have been used to measure IR, including fasting insulin, homeostatic model assessment for IR (HOMA–IR), and quantitative insulin sensitivity check index (QUICKI) [40, 41]. A previous study demonstrated a significant association between the HOMA–IR and CVD risk [42]. However, these fasting insulin-based tools face challenges in their practical application in clinical settings because of difficulties associated with directly measuring insulin levels. Therefore, novel non-insulin-based IR markers, including the three markers used in this study, have been suggested to address these limitations.

AIP, proposed in 2000, is an atherogenic index calculated as the logarithm of the ratio of triglycerides to HDL cholesterol [43]. It has been extensively studied as an independent risk marker for CVD [16, 17, 44]. The TyG index, reflecting the combined effect of triglycerides and fasting plasma glucose, is associated with early IR and related metabolic abnormalities [45–47]. Indeed, recent epidemiological studies have reported that the TyG index is more valuable than the HOMA–IR index in assessing IR and serves as a key indicator for screening metabolic syndrome [47, 48]. Furthermore, one study highlighted the advantage of the TyG index in evaluating peripheral IR in non-obese Koreans compared to Western populations [49]. The METS–IR, a composite of several variables including fasting glucose, fasting triglycerides, BMI, and HDL-C, demonstrated a significant correlation with the euglycemic–hyperinsulinemic clamp, the gold standard method for measuring insulin sensitivity [50]. In addition, it showed promising results in predicting cardiometabolic risks, including hypertension and T2DM [51, 52].

Several mechanisms have been proposed to support the idea that non-insulin-based IR indices can accurately represent IR. Notably, the role of TG was emphasized for all three markers used in this study. Excessive caloric intake and insufficient physical activity lead to storing surplus energy, such as TG, in the liver and other tissues. This process of carbon energy storage appears to be a protective mechanism against the cytotoxic accumulation of fatty acids (FA). However, hepatic IR is associated with the accumulation of TG and FA metabolites. Although this short-term protection from FA sequestration may be beneficial, it can lead to long-term complications, including the development of further IR, eventually progressing to T2DM [53].

Similarly, pancreatic islet cells experience impaired glucose metabolism and beta cell dysfunction owing to triglyceride overload. Moreover, elevated levels of FA and glucose lead to the accumulation of esterified FA metabolites, contributing to islet cell dysfunction. In the context of skeletal muscle-related IR, characterized by peripheral IR, hypertriglyceridemia has been known to impede insulin activity within the muscle and hinder glucose uptake [54]. This condition leads to myosteatosis and induces the production of inflammatory cytokines, ultimately leading to muscle catabolism [55]. Previous studies have demonstrated a notable association between METS–IR, a novel marker including anthropometric measurements, and visceral adiposity, encompassing intrahepatic and intrapancreatic fat content [50].

Glucose and lipid metabolism are tightly interconnected; both play essential roles in energy metabolism and are regulated by the liver. T2DM has a characteristic dyslipidemia known as the lipid triad, which includes elevated TG, low HDL-C, and small dense low-density lipoprotein [56–58]. The relationship between lipids and glucose is bi-directional. Considering this relationship, it is evident why HDL is included as a component of IR markers, such as AIP and METS–IR.

Our results were consistent with those of previous studies; however, there were some discrepancies. Our findings indicate that AIP does not show significant predictive capability for the occurrence of CVD. Cai et al. conducted a hospital-based observational study on young adults (≤ 35 years) undergoing coronary angiography to investigate the link between AIP and the presence and severity of acute coronary syndrome (ACS). A sex-based subgroup analysis found an independent association between the AIP and ACS risk in males [59]. Conversely, a case–control study conducted in China through propensity score matching, which included 348 postmenopausal CAD cases and 348 controls, revealed that the AIP could potentially function as a robust predictor of CAD risk in postmenopausal women [60]. Based on the conflicting findings of previous research, particularly in terms of sex disparities, further investigation is warranted to elucidate the sex-specific implications of AIP.

Furthermore, an analysis comparing the three markers revealed that METS–IR and TyG exhibited comparable significance as predictive markers for CVD. Wu et al. investigated the associations of TG/HDL-C ratio, TyG index, and METS–IR with CAD presence and severity in 802 patients undergoing coronary angiogram (CAG) for suspected CAD. Their findings revealed that the TG/HDL-C ratio and METS–IR independently predicted the presence of CAD, and METS–IR emerged as the sole predictor of CAD severity [61]. Although the investigations above included a risk-specific group that underwent CAG, this study encompassed a broader general population.

Another possibility is that when comparing the constituents of these markers, elements such as fasting plasma glucose and BMI might play a more significant role in predicting CVD risk within an East Asian population. This hypothesis emphasizes the importance of detecting and managing impaired fasting glucose and obesity in advance and underscores the need for targeted interventions encompassing nutritional, behavioral, and pharmaceutical approaches within the Korean population.

This study is the first to compare novel non-insulin-based markers for predicting CVD risk in the general population. Our findings indicate that the TyG index and METS–IR have similar predictive capabilities for CVD, suggesting their potential as valuable risk stratification markers for CVD, particularly in East Asian populations. The results of this study have the potential to guide the selection of personalized markers, and integrating these markers with established risk factors is anticipated to enhance their predictive efficacy. However, it is important to note that predictive values can differ among populations and settings. Further research is required to validate these results across diverse populations and to investigate personalized cutoff value recommendations considering factors, such as sex and ethnicity.

This study has strengths, because it engaged in a prospective cohort analysis encompassing many Korean participants with connections to HIRA data derived from the country's universal coverage system. Consequently, the potential for data gaps is minimal [62]. However, this study had limitations. First, the HERAS–HIRA data set focused solely on newly developed CVD cases and lacked information on calcium scores or coronary angiography. It is important to note that the outcome of the KoGES cohort was based on self-reported CVD cases, which may consequentially affect the study's findings. Future studies should consider subgroup evaluation according to CVD type after data accumulation. In addition, The HERA–HIRA cohort participants via health check-ups are usually interested in their health problems, leading to selection bias. Finally, this study did not consider important confounding variables, such as the family history of CVD, lifestyle factors, and comorbidities, which could have influenced the observed relationships between IR markers and CVD.

## Conclusions

Among the three large cohorts of Korean men and women, three novel surrogate markers of IR were positively correlated with the prevalence of CVD. The TyG index and METS–IR showed similar predictive values, whereas AIP had a lower significance in predicting CVD.

## Data Availability

The data sets used and/or analyzed in the current study are available from the corresponding author upon reasonable request.

## Abbreviations

ACS: Acute coronary syndrome
AIP: Atherogenic index of plasma
BMI: Body mass index
CAD: Coronary artery disease
CAG: Coronary angiogram
CVD: Cardiovascular disease
eGFR: Estimated glomerular filtration rate
HDL-C: High-density lipoprotein cholesterol
HERAS–HIRA: Health risk assessment study and Korea health insurance review and assessment
HRs: Hazard ratios
IR: Insulin resistance
KoGES: Korean genome and epidemiology study
LDL-C: Low-density lipoprotein cholesterol
METS–IR: Metabolic score for insulin resistance
TG: Triglyceride
TyG index: Triglyceride–glucose index
